# Fabrication of Eco-Friendly Hydrolyzed Ethylene–Maleic Anhydride Copolymer–Avermectin Nanoemulsion with High Stability, Adhesion Property, pH, and Temperature-Responsive Releasing Behaviors

**DOI:** 10.3390/molecules29051148

**Published:** 2024-03-05

**Authors:** Yuxin Cheng, Zeyu Pan, Liming Tang, Yanbin Huang, Wantai Yang

**Affiliations:** Key Laboratory of Advanced Materials of Ministry of Education of China, Department of Chemical Engineering, Tsinghua University, Beijing 100084, China; chengyx22@mails.tsinghua.edu.cn (Y.C.);

**Keywords:** ethylene–maleic anhydride copolymer, avermectin, phase inversion emulsification, response surface methodology, nanopesticide

## Abstract

In this study, novel amphiphilic polymer emulsifiers for avermectin (Avm) were synthesized facilely via the hydrolysis of ethylene-maleic anhydride copolymer (EMA) with different agents, and their structures were confirmed by various techniques. Then, water-based Avm-nanoemulsions were fabricated with the emulsifiers via phase inversion emulsification process, and superior emulsifier was selected via the emulsification effects. Using the superior emulsifier, an optimal Avm-nanoemulsion (defined as Avm@HEMA) with satisfying particle size of 156.8 ± 4.9 nm, encapsulation efficiency (EE) of 69.72 ± 4.01% and drug loading capacity (DLC) of 54.93 ± 1.12% was constructed based on response surface methodology (RSM). Owing to the emulsifier, the Avm@HEMA showed a series of advantages, including high stability, ultraviolet resistance, low surface tension, good spreading and high affinity to different leaves. Additionally, compared to pure Avm and Avm-emulsifiable concentrate (Avm-EC), Avm@HEMA displayed a controlled releasing feature. The encapsulated Avm was released quite slowly at normal conditions (pH 7.0, 25 °C or 15 °C) but could be released at an accelerated rate in weak acid (pH 5.5) or weak alkali (pH 8.5) media or at high temperature (40 °C). The drug releasing profiles of Avm@HEMA fit the Korsmeyer–Peppas model quite well at pH 7.0 and 25 °C (controlled by Fickian diffusion) and at pH 7.0 and 10 °C (controlled by non-Fickian diffusion), while it fits the logistic model under other conditions (pH 5.5 and 25 °C, pH 8.5 and 25 °C, pH 7.0 and 40 °C).

## 1. Introduction

In agriculture, the use of pesticides provides effective pest control and improves crop yields. It has been reported that pesticides contribute up to 50% of crop gain in developing countries. However, the active ingredients of pesticides are mostly organic compounds insoluble in water, and the traditional pesticide formulations are easily lost during application due to volatilization, leaching, runoff, degradation and photolysis, resulting in low pesticide utilization efficiency [1,2]. In recent years, nanopesticides, which are applied in the form of nanoemulsions [3], have gradually been applied in the agricultural field due to their ability to address the inefficiencies of pesticides, environmental pollution, bioaccumulation, and pest resistance [4]. Nanopesticides have kinetic stability and can significantly improve the solubility and bioavailability of the encapsulated pesticide [5], playing an increasingly significant role in many hydrophobic pesticide-based nanoformulations.

Owing to their tunable functionalization, diverse structures, environmental friendliness, and emulsification ability, amphiphilic polymers have stimulated the interest of researchers in nanopestcides recently [6,7]. So far, the application of amphiphilic polymers in the field of nanopesticides lacks theoretical basis, and the selection of polymer emulsifiers faces significant randomness and uncertainty. It is still a challenge to seek an effective amphiphilic polymer emulsifier capable of forming nanoemulsions of highly hydrophobic pesticides. The amphiphilic polyolefin copolymers [8,9,10,11,12,13] derived from styrene maleic anhydride copolymer (SMA) have the characteristics of easy functionalization, straightforwardness, great biocompatibility, low cost, and can form self-assembled micelles in water to encapsulate various objects, including drugs [14,15,16]. However, SMA derivatives have rarely been used to form nanopesticides. For example, only micron sized diflubenzuron suspension concentrate with low stability was constructed using a polystyrene–maleic anhydride sulfonic acid sodium emulsifier [17]. Compared to SMA, ethylene–maleic anhydride copolymer (EMA) shows higher reactivity and higher economic benefits, and its hydrolytic product [18] can have higher hydrophilicity than SMA counterpart. Thus, they should be potential candidates for emulsifying highly hydrophobic pesticides. In the past several decades, EMA and its modified products have been explored as virus inhibitors [19], immunosorbents [20], enzyme and bioactive protein support [21], and bactericide support [22] but still not as emulsifiers of pesticides yet.

Among all kinds of pesticides, avermectin (Avm) is one of the most important categories for its effectiveness in animal husbandry and human parasitic diseases. Avm has a direct contact killing effect on predatory insects and parasitic natural enemies, but its damage to beneficial insects is minimal due to the low residue on the plant surface. Avm is adsorbed by soil and does not move and is decomposed by microorganisms, so it has no cumulative effect in the environment [23,24,25]. However, Avm is a natural macrolide with extremely low solubility. Moreover, Avm is susceptible to decomposition in the presence of ultraviolet (UV) light and conventional Avm formulations exhibit abrupt initial releasing behavior and low utilization [26]. Therefore, it is of great significance to develop highly efficient and eco-friendly water-based Avm-nanoemulsion, which could improve utilization, reduce volatilization losses and photodegradation as well as achieve controlled drug release, adapting to the internal environment of insects. Especially, the controlled stimuli-responsive behaviors of drug releasing have drawn researchers’ attention in many fields for improving drug bioavailability. The stimulation factors could be pH [27], temperature [28], enzyme [29], redox [30], magnetic [31], ultrasound [32], and so on. Among them, pH- and temperature-responsive characteristics are of better value in practical applications for nanopesticides because most of the pests possess weakly acidic or weakly alkaline body fluids and the growth of pest populations is usually sensitive to the temperature. Nevertheless, most preparation methods of pH- or temperature-responsive nanoemulsions are complicated and their performances need to be improved.

Moreover, there are many component and process variables in the fabrication of pH and temperature-responsive Avm-nanoemulsions. Some reports [33,34,35,36] indicated that the characteristics (polarity, water solubility, viscosity, density, etc.) of the oil phase and the type and concentration of components present in the aqueous phase may affect the physicochemical properties of the final formulation. A simpler and more convenient method is urgently needed to rationally select the composition of stable Avm-nanoemulsions with idealized properties [37] compared to the complex and challenging ternary phase diagram during various phase inversion emulsification processes. Recently, response surface methodology (RSM) [38,39,40,41] was applied to determine the optimal composition and operating conditions for the preparation of various nanoemulsions. The RSM should be valuable for efficiently and quickly selecting the optimal formulation in the fabrication of nanopesticides.

In this study, in order to form an Avm-nanoemulsion with controllable releasing features, we prepared two novel amphiphilic polycarboxylate emulsifiers via the hydrolysis of EMA through simple and efficient processes. Based on these emulsifiers, eco-friendly water-based pH and temperature-responsive Avm-nanoemulsions were fabricated via the phase inversion emulsification technology. The optimal formulation of the Avm-nanoemulsion (Avm@HEMA) was selected with RSM according to the particle size, encapsulation efficiency and drug loading. Additionally, the stability, adhesion property and sustained releasing properties at different pH and temperatures of Avm@HEMA were investigated. The cumulative releasing profiles of Avm@HEMA were investigated via fitting with some typical releasing kinetic models. Because of the emulsifier, the obtained Avm@HEMA exhibits a series of desired performances, including high encapsulation efficiency, high drug loading, good stability, strong affinity to leaves, controlled releasing features. Therefore, this study should inspire more effective designs of multifunctional polymer emulsifiers for the fabrication of advanced nanopesticides.

## 2. Results and Discussion

### 2.1. Preparation of Emulsifier and Fabrication of Avm-Nanoemulsion

Typically, in the fabrication of a pesticide nanoemulsion via the phase inversion emulsification technique, an emulsifier and a pesticide are first dissolved in a solvent to form an oil phase, which is then added dropwise into water under stirring to form the nanoemulsion. From the application viewpoint, key parameters that influence the fabrication process, such as the type of emulsifier, the weight ratio of oil phase and water phase, the weight ratio of oil phase and emulsifier, as well as the type and content of oil phase solvent, should be defined quickly with fewer trials [42] to obtain an optimal formulation.

**Scheme 1:**
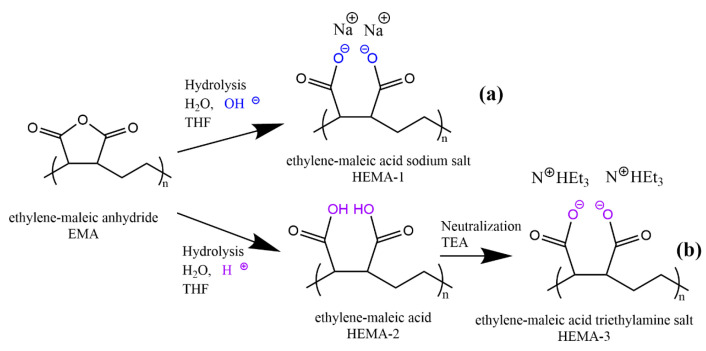
Synthetic process of (**a**) HEMA-1 and (**b**) HEMA-3.

In order to fabricate an Avm-nanoemulsion via the phase inversion emulsification process, two new polymer emulsifiers were designed and prepared via different approaches, one is the alkali hydrolysis of EMA by using sodium hydroxide (see Scheme 1a) to generate the first emulsifier (defined as HEMA-1) bearing sodium carboxylate groups, and the other is the acid hydrolysis of EMA by using hydrochloric acid to give ethylene-maleic acid copolymer (defined as HEMA-2), which was further neutralized with TEA to generate the second emulsifier (defined as HEMA-3) (see Scheme 1b) bearing ammonium carboxylate groups. With both hydrophobic carbon chain backbone and hydrophilic carboxylate side groups, the obtained amphiphilic polymers, HEMA-1 and HEMA-3, should be potential emulsifiers of pesticides. In particular, with a rather high weight percent (80.6%, calculated based on HEMA-2) of maleic acid units, the obtained polymer emulsifiers are expected to have excellent emulsifying capability for very hydrophobic Avm, conducive to forming its nanoemulsion in water.

**Figure 1:**
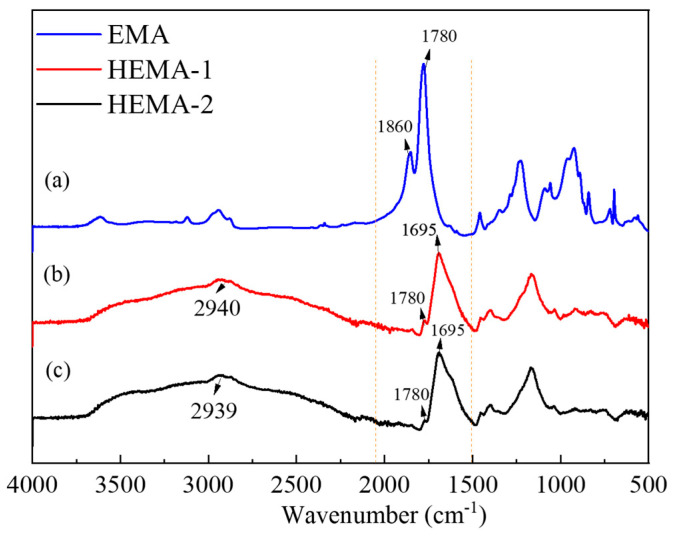
FT-IR spectra of (**a**) EMA; (**b**) HEMA-1; (**c**) HEMA-2.

The FT-IR and ^1^H NMR spectrometers were applied for characterizing the molecular structures of EMA, HEMA-1, and HEMA-2. The FT-IR spectra in Figure 1 indicate that the infrared absorption variation in the spectrum is concentrated at 1600–1860 cm^−1^ and 2500–3500 cm^−1^. Figure 1a shows the absorption peaks at 1780 cm^−1^ and 1860 cm^−1^, respectively, belonging to –C=O– of the maleic anhydride ring in EMA [43]. After the hydrolysis of EMA with sodium hydroxide or hydrochloric acid, it is indicated by Figure 1b,c that the peaks at 1780 cm^−1^ and 1860 cm^−1^ are weakened or even absent, while the absorption peak of the stretching vibration at 1695 cm^−1^ for –C=O of maleic acid unit is significantly enhanced, demonstrating that the ring of maleic anhydride unit of EMA was opened in the hydrolysis reaction, forming both HEMA-1 and HEMA-2 [44]. However, the stretching vibration peaks at 1226 cm^−1^ and 1160 cm^−1^ for –C–O–C– of maleic anhydride unit still existed, indicating that the degree of hydrolysis reaction did not reach 100%, in agreement with the literature result [43]. The ^1^H NMR spectra of HEMA-1 and HEMA-2 in Figure S1 both show a new peak of –COOH at 12.13 ppm after the hydrolysis reaction compared to EMA, indicating the existence of maleic acid unit in them [45], which was formed via the hydrolysis of the maleic anhydride unit in EMA, in agree with the result of the FT-IR spectra. Moreover, the local magnification ^1^H NMR spectrum of EMA in Figure S2 confirms that EMA is an alternating copolymer due to the fact that the integral area ratio of hydrogen atoms of ethylene (peak a–d) and maleic anhydride (peak e,f) is 1.00:1.04.

To calculate the hydrolysis degree, the molecular weight and element content of HEMA-1 and HEMA-2 were determined with the results shown in Table 1. The GPC analysis result indicates that the number average molecular weight (M_n_), weight average molecular weight (M_w_), and polydispersity index (PDI) of both HEMA-1 and HEMA-2 showed some changes compared to EMA, which might be attributed to the increase in the actual molecular weight after the hydrolysis reaction and an increase in the residence time due to the increase in molecular polarity after the conversion of anhydride to carboxyl group. Meanwhile, the elemental analysis result reveals that both HEMA-1 and HEMA-2 have much improved oxygen content compared to EMA, indicating the formation of two carboxyl groups from one anhydride group. On the basis of the oxygen content, the hydrolysis degree could be calculated to be 83.56% and 83.34% for HEMA-1 and HEMA-2, respectively, indicating that both approaches gave almost the same hydrolysis degree. The rather high hydrolysis degree reveals that the desired products were obtained.

**Figure 2:**
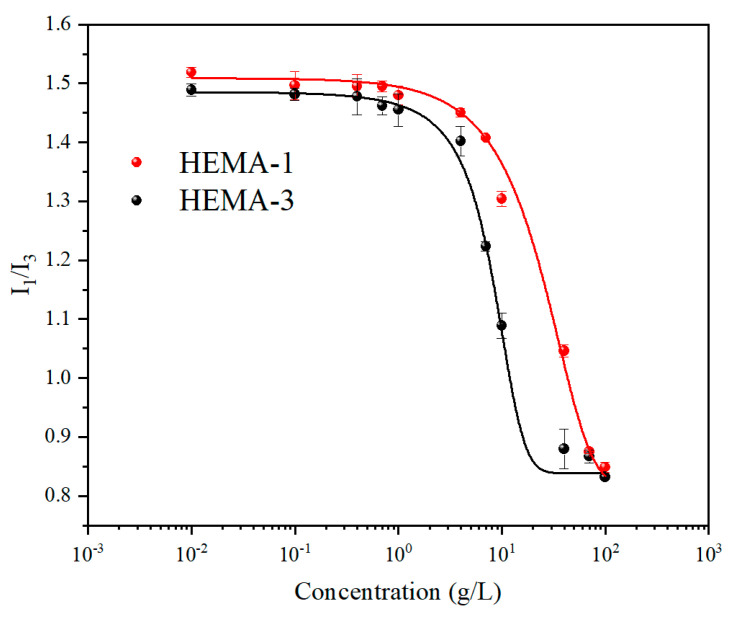
The I_1_/I_3_ values of pyrene at different concentrations of the emulsifiers in aqueous solutions.

In the phase inversion emulsification process, following the addition of the oil phase into water, the emulsifier molecules assembled into particles with the pesticide molecules under the driving of hydrophobic interactions. Meanwhile, the hydrophilic ionized carboxyl groups of the emulsifier molecules cover the surface of the particles, providing electrostatic repulsion for stability. To understand the emulsifying performance of the two emulsifiers, the fluorescence spectrometer was used to determine the critical micelle concentration (CMC) of them. The CMC can be determined by the I_1_/I_3_ value, which is the ratio of the fluorescence intensity (I_1_) of the first electron vibration peak at 373 nm to that (I_3_) of the third electron vibration peak at 384 nm in the spectrum, using pyrene as a fluorescent probe [43]. The I_1_/I_3_ value as a function of the concentration of HEMA-1 or HEMA-3 is illustrated in Figure 2. The fitted curves of HEMA-1 and HEMA-3 receive correlation coefficients of 0.9982 and 0.9976, respectively. On the basis of the method reported by Aguiar [46,47], the CMC values of HEMA-1 and HEMA-3 were calculated to be 16.0 g/L and 7.5 g/L, respectively, indicating that HEMA-3 could form micelle at a much lower concentration compared to HEMA-1.

In a preliminary formulation study (0.125 g HEMA-1 or HEMA-3, 0.25 g Avm, 1.0 g DMSO, and 10.0 g H_2_O), two Avm-nanoemulsions were formed via phase inversion emulsification technology using HEMA-1 and HEMA-3 as the emulsifiers separately. From the results in Table 2, the nanoemulsion of HEMA-3 gives the Z-average particle size of 231.0 nm and PDI of 0.069, smaller than the Z-average particle (563.1 nm) and PDI (0.109) of the nanoemulsion of HEMA-1. The Zeta potential of both nanoemulsions is less than −30 mV, indicating that the particles are stabilized by the electrostatic repulsion force. Moreover, the nanoemulsion of HEMA-3 has a Zeta potential of −50.4 mV, smaller than that (−32.0 mV) of the nanoemulsion of HEMA-1. Therefore, HEMA-3 is superior to HEMA-1 according to the emulsification results of the nanoemulsions, and the following investigations are all based on HEMA-3 unless otherwise stated.

**Figure 3:**
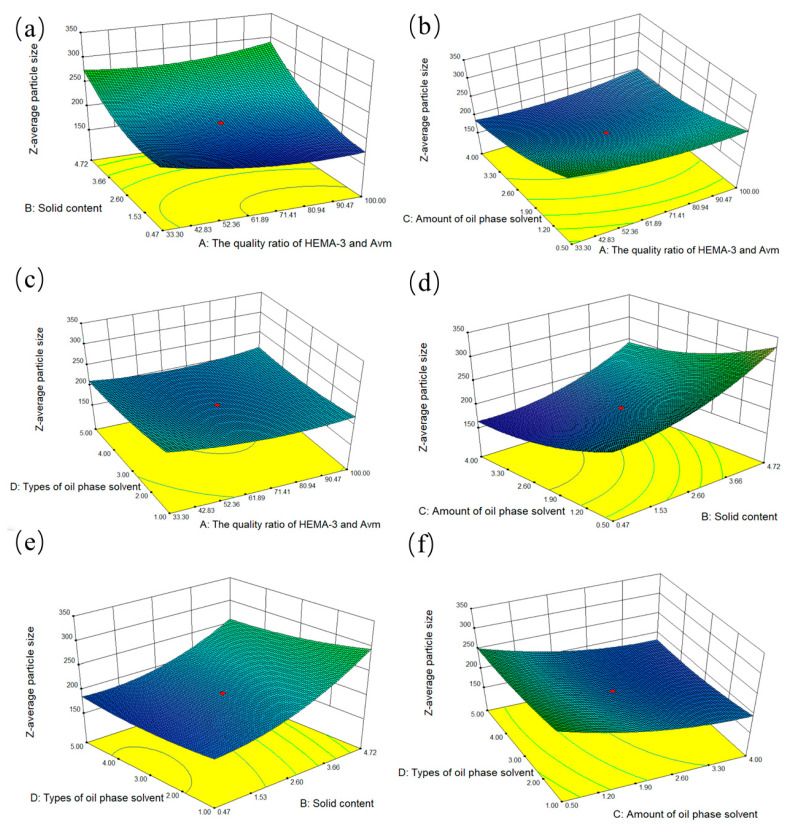
Response surface and contour plots of the interactions between (**a**) A vs. B, (**b**) A vs. C, (**c**) A vs. D, (**d**) B vs. C, (**e**) B vs. D, and (**f**) C vs. D on the Z-average particle size of Avm-nanoemulsion.

In order to obtain the optimal formulation, the effects of the weight ratio of HEMA-3 and Avm (33.33–100%, *w*/*w*, A), solid content (0.5–5%, *w*/*w*, B), amount of oil phase solvent (0.5 g–4 g, C), and the type of oil phase solvent (selected from NMP, acetonitrile, DMF, DMSO, and DMPU, using number 1–5, respectively to represent them, D) on three response variables, including Z-average particle size (Y_1_), encapsulation efficiency (EE) (Y_2_) and drug loading capacity (DLC) (Y_3_) of Avm-nanoemulsions, were analyzed using four-factor RSM with 5 levels of central composite design (CCD) each using Design Expert software (version 8.0.6) [38,45], as indicated in Table S1. Surface responses of the quadratic polynomial model of Z-average particle size and liner models of EE or DLC are generated by varying two of the four independent variables within the experimental range while holding the other two constants at the central points. Based on the fact that the *p*-value (the *p*-value is used in hypothesis testing to help decide whether to accept the null hypothesis. The more significant the *p* value, the more likely to accept the null hypothesis) is significant and the lack-of-fit value of these models provided by Design Expert software is not significant, these models are appropriate. The goodness of fit of the model is evaluated by coefficient determination (R^2^) [41]. The significant differences between independent variables are determined by ANOVA. Response surfaces and 3D contour plots of the fitted equations are generated to visualize the interaction effect of the independent variables on responses better in Figure 3, Figures S3 and S4. The optimal formulation is obtained by comparing the experimental values with the predicted values.

The *p*-value in Figure 3 indicates that the solid content (B) and the amount of oil phase solvent (C) have a stronger influence on the Z-average particle size of Avm-nanoemulsion than the other independent variables. The interactions are the two occurring between the weight ratio of HEMA-3 and Avm (A) and the solid content (B) as well as the weight ratio of HEMA-3 and Avm (A) and the amount of oil phase solvent (C). As indicated in Figure 3a, when the solid content is kept at a fixed level, the Z-average particle size decreases and then increases as the weight ratio of HEMA-3 and Avm (A) increased, with a minimum value in the range from 52.36% to 71.41%, due to the fact that a suitable concentration of HEMA-3 is required for the formation of nanoemulsions. At a lower concentration of HEMA-3, the nanoemulsion does not form a complete stabilizing surface layer, resulting in agglomeration [48,49]. While at a higher concentration of HEMA-3, the bridging of the emulsifier molecules or the formation of empty micelles can lead to flocculation or the Ostwald ripening [50]. However, when the weight ratio of HEMA-3 and Avm (A) is fixed, the Z-average particle size hardly changes in the range of 0.5–3% for the solid content (B). When the solid content (B) increases, the Z-average particle size rises sharply, mainly due to the increased probability of collision of particles in Brownian motion, leading to agglomeration. In contrast, from Figure 3b, it can be seen that the amount of the oil phase solvent (C) has a negative effect on the Z-average particle size. As the amount of the oil phase solvent (C) increases, the Z-average particle size decreases rapidly and then changes slightly because the Ostwald ripening tends to occur at less oil phase solvent values and the emulsions coarsen with larger particle sizes, leading to flocculation, aggregation, and phase separation at a later stage [51]. As the solvent content of the oil phase increases, the overall emulsification effect of Avm becomes better and the Z-average particle size tends to be smaller.

For EE and DLC in Figures S3 and S4, the *p*-value indicates that the weight ratio of emulsifier and Avm (A) has the strongest influence on both EE and DLC of Avm-nanoemulsion, while the other variables and interactions hardly change the tendency of EE or DLC. From the results in Figures S3 and S4, when the weight ratio of HEMA-3 and Avm (A) increases from 61.89% to 100%, the EE is at above 60%, which is within the acceptable range. Figure S3a shows when the weight ratio of emulsifier and Avm (A) is between 33.33% and 61.89%, the DLC decreases slowly. However, as the solid content (B) increases and the amount of oil phase solvent (C) decreases, the change in the trend of DLC is not obvious, as indicated in Figure S3c. The above result is also demonstrated in Figures S3 and S4. Therefore, apart from the weight ratio of HEMA-3 and Avm (A), the other independent variables do not respond significantly to either EE or DLC.

Numerical optimization of the formulation of a nanoemulsion loaded with Avm is carried out through Design Expert software using the desirability function. The optimal formulation is expected to be those leading to a stable emulsion with satisfying Z-average particle size, EE, and DLC. The optimal formulation with 100% desirability is predicted for the weight ratio of HEMA-3 and Avm (A) 55.84% (*w*/*w*), the solid content (B) 2.38% (*w*/*w*), the oil phase solvent (C) 3.92 g, as well as the type of oil phase solvent DMSO (D), with a Z-average particle size of 161.3 nm, an EE of 63.70%, and a DLC of 60.18%. From a practical point of view, the Avm-nanoemulsion with a higher solid content is more desirable in terms of transport and storage. For environmental and safety reasons, as an aqueous emulsion system, the lower the oil phase solvent content, the better. Further considering the above factors, the optimal formulation was obtained, which included the weight ratio of HEMA-3 and Avm (A) at 57.14% (*w*/*w*), the solid content (B) at 2.74% (*w*/*w*), 1 g oil phase solvent (C), and using DMSO (D) as the oil phase solvent. Then, three experimental replicates were made with the optimal formulation, the obtained Avm-nanoemulsion (defined as Avm@HEMA) had a Z-average particle size of 156.8 ± 5.0 nm, a PDI of 0.078 ± 0.003, a Zeta potential of −49.2 ± 0.9 mV, EE of 69.72 ± 4.01%, DLC of 54.93 ± 1.12%, and solid content of 3.03 ± 0.01%. As expected, these results are close to those obtained at lower solid content of 2.38%. Unless otherwise stated, the subsequent investigations are all based on the optimal nanoemulsion, Avm@HEMA.

### 2.2. Morphology of Avm@HEMA

**Figure 4:**
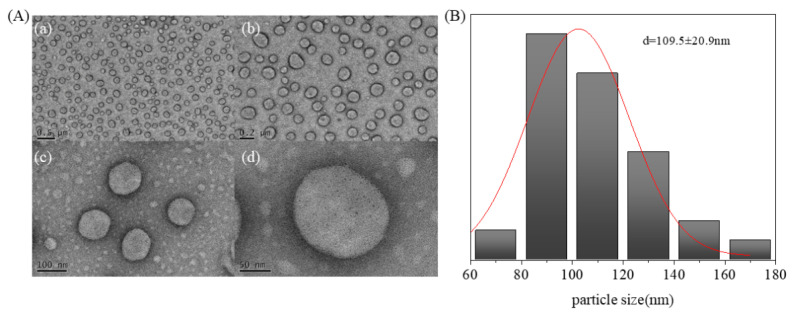
(**A**) TEM images of the particles in Avm@HEMA under different scale bars: (**a**) 0.5 μm; (**b**) 0.2 μm; (**c**) 100 nm; (**d**) 50 nm, and (**B**) particle size distribution based on TEM image using Nano Measurer 1.2 software.

As indicated above, the optimal nanoemulsion Avm@HEMA was fabricated through a facile and eco-friendly process. Then, the obtained nanoemulsion was subjected to TEM observation. From Figure 4A, approximately spherical particles are observed in all the images. The particles give a statistical average particle size of 109.5 ± 20.9 nm (see Figure 4B), smaller than that (156.9 ± 5.0 nm) tested by DLS. This is consistent with our previous result [27]. Moreover, Figure S5 is the confocal laser scanning microscopic image of Avm@HEMA with Nile red labeled. Similar to the TEM result, there are many red nanoscale particles in the sample, indicating that Avm molecules have been encapsulated in the nanoparticles.

### 2.3. Stability of Avm@HEMA

**Figure 5:**
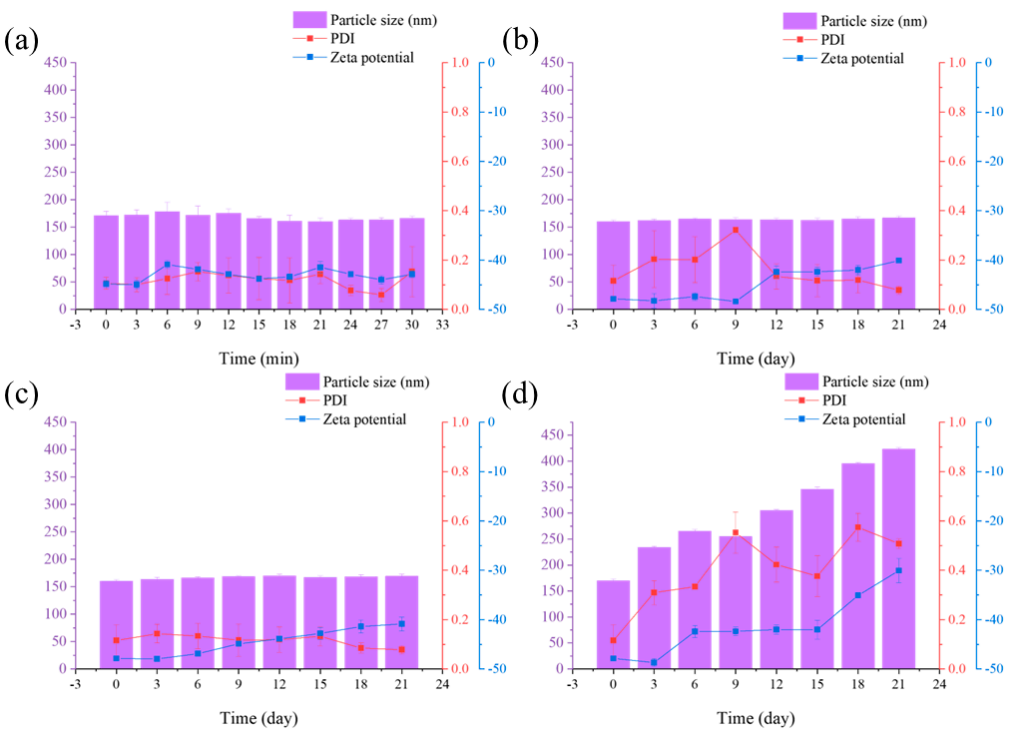
Changes in Z-average particle size, PDI, and Zeta potential of Avm@HEMA after (**a**) centrifugation at 10,000 rpm; (**b**) storage at 0 ± 2 °C; (**c**) storage at 25 ± 2 °C; (**d**) storage at 54 ± 2 °C.

Stability is an important index for Avm-nanoemulsion from a practical viewpoint [52]. In this study, the centrifugal stability, physical stability, dilution stability, storage stability, and light stability of Avm@HEMA were tested. Firstly, after accelerated sedimentation treatment of the nanoemulsion by centrifugation at 10,000 rpm, there was only a small increase in the transparency in the nanoemulsion without delamination and precipitation. Figure 5a shows that during the 30 min centrifugal treatment, the Z-average particle size is maintained in the range of 175 nm–200 nm, the PDI remains at around 0.1, and the Zeta potential is kept between −40 mV–−50 mV, indicating a high centrifugal stability of the nanoemulsion.

Next, the thermal stability of Avm@HEMA was evaluated by storage at different temperatures for different periods. Figure 5b–d shows the changes in Z-average particle size, PDI, and Zeta potential of Avm@HEMA at 0 ± 2 °C (b), 25 ± 2 °C (c), and 54 ± 2 °C (d) respectively. The results show that during the long-term storage at low (0 °C) or room temperature (25 °C) for 21 days, the nanoemulsions maintained a particle size of about 160 nm. There is only a slight change in the PDI (mostly still below 0.3), and the Zeta potential is kept between −40–−50 mV, indicating that the Avm-nanoemulsions maintained high stability at low and room temperatures, probably due to the kinetic stability of the nanoemulsion and the stabilizing effect of the emulsifier interfacial film. However, after 21 days of storage at 54 °C, a few visible precipitates appeared in the sample, the Z-average particle size expanded from 156.9 nm to 423.5 nm, the PDI increased from 0.078 to 0.510, and the Zeta potential increased from −50 mV to −30 mV, indicating a decrease in the homogeneity and stability. The decreased stability may be attributed to the fact that the moving and dissolving of the emulsifier molecules from the particles was accelerated, and the hydrophobic core was significantly swollen at high temperature [27]. The increased PDI and Zeta potential may weaken the Brownian motion, enhance Ostwald ripening along with the particle agglomeration in the emulsion, and dramatically decrease the stability [53].

In the practical application, the nanoemulsion should be diluted before being sprayed in the field. The dilution stability of the nanoemulsion (with a 1.9% active ingredient content) was tested by diluting it 20 times, 200 times, and 2000 times using deionized water. Figure S6a shows that the transparency of Avm@HEMA increases significantly as the dilution times increased. However, the Z-average particle size, PDI, and Zeta potential remained almost intact after dilution. Moreover, after one month storage at room temperature, Avm@HEMA still remained homogeneous and unstratified, as shown in Figure S6b and Table S2. Meanwhile, it showed the Z-average particle size of 163.7 ± 2.9 nm, the PDI of 0.133 ± 0.021, and the Zeta potential of −44.7 ± 0.3 mV, which are almost the same as those before storage, showing the long-term storage stability.

Avm is a kind of photosensitive pesticide. Encapsulated in suitable nanoparticles should be an effective way to retard the degradation of Avm. Figure S7 shows that as the UV light exposure time prolonged, the degradation percentage of Avm in Avm@HEMA is much lower than that of pure Avm. After 20 h of UV light exposure, only 38.21% Avm remained for pure Avm sample, while 69.75% Avm remained for Avm@HEMA. This result indicates that in the UV light exposure period, the outer layer of the particles could absorb, scatter and reflect UV light, providing shielding effect to the inner Avm molecules.

In sum, the above results demonstrated the satisfying stability of the nanoemulsion, which should make it suitable for agriculture applications.

### 2.4. Wetting and Adhesion Behavior of Avm@HEMA on Leaves

High affinity, effective deposition and strong adhesion of a pesticide nanoemulsion to plant leaf can facilitate the spreading and wetting on the leaf surface, which is necessary to prevent droplet slippage, reduce waste and prolong the effective time of the pesticide. Firstly, the surface tension of the aqueous solution of HEMA-3 at different concentrations was tested. From the result in Figure S8, it can be seen that the surface tension decreases significantly with the increasing concentration of HEMA-3 in the low concentration range, after passing through a critical point, the surface tension is finally stabilized at 35.9 ± 0.2 mN/m. According to the critical point, the CMC value (7.8 g/L) of HEMA-3 could be obtained, which is quite close to that (7.5 g/L, see Figure 2) determined by the fluorescence method. Using deionized water (surface tension = 71.0 mN/m) as a control, the contact angles (CA) of Avm-EC (surface tension = 55.3 ± 0.8 mN/m) and Avm@HEMA (surface tension = 49.3 ± 0.4 mN/m) on the leaves of rice (surface tension = 29.9 mN/m), cabbage (surface tension = 38.9 mN/m), and cucumber (surface tension = 58.7 mN/m) at field application concentration (0.02 wt%) are shown in Figure S9. It is noticed that the CA of Avm@HEMA on these leaves are 57.2 ± 1.0°, 49.4 ± 1.3°, and 40.1 ± 1.7° respectively, lower than those of Avm-EC and water, demonstrating the excellent spreading of Avm@HEMA on the leaves. Comparing the CA of the same column in Figure S9, it is clear that the leaf with a larger surface tension turned to result in a lower CA for the same measuring liquid.

**Figure 6:**
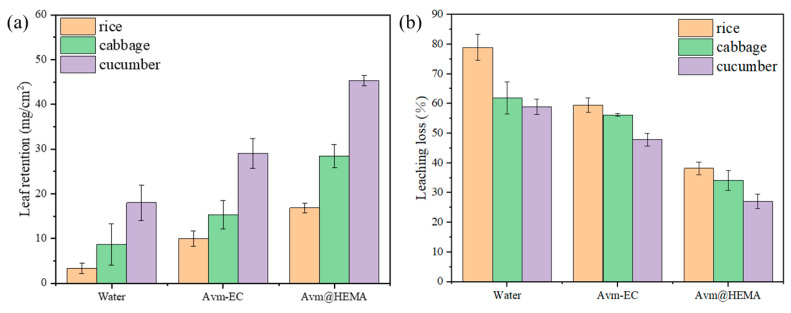
(**a**) Leaf retention of water, Avm-EC, and Avm@HEMA on various leaves; (**b**) leaching loss of water, Avm-EC, and Avm@HEMA on various leaves.

Then, the leaf retention was measured to understand the affinity of pesticide liquids on the leaves. Figure 6a shows that the leaf retention decreases for all the liquids by the order of cucumber, cabbage and rice. The low retention on rice and cabbage leaves are mainly because both of the two leaves have waxy structures that reduce the affinity with the liquids [54]. For the same leaf, the leaf retention decreases by the order of Avm@HEMA, Avm-EC, and water, just opposite to the trend of the surface tensions of the liquids. Moreover, the leaching loss on the leaves of various liquids were further determined. From the results in Figure 6b, it is noticed that the leaching loss is just opposite to that of leaf retention, i.e., the higher the leaf retention, the lower the leaching loss. For the same leaf, Avm@HEMA exhibits the lowest leaching loss among all the liquids. The above results indicate that owing to the low surface tension providing by the emulsifier, Avm@HEMA displays good droplet spreading, wetting ability, and strong affinity for both hydrophilic and hydrophobic leaves, demonstrating its potential application in the field.

**Figure 7:**
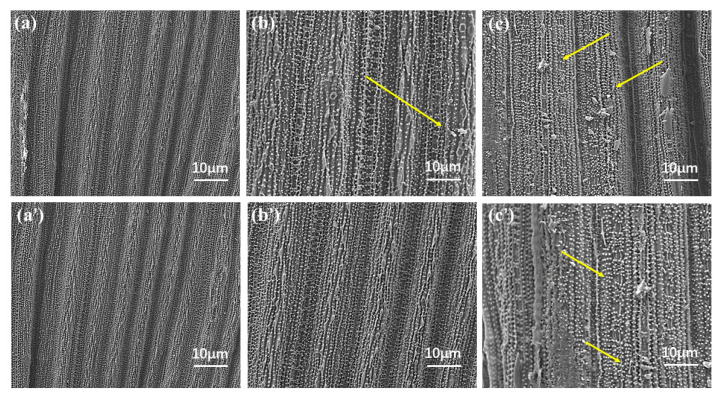
SEM images of rice leaf with water and different Avm formulations. (**a**) before and (**a’**) after leaching for water; (**b**) before and (**b’**) after leaching for Avm-EC; (**c**) before and (**c’**) after leaching for Avm@HEMA. (The arrows point to Avm nanoparticles remaining on leaves.)

**Figure 8:**
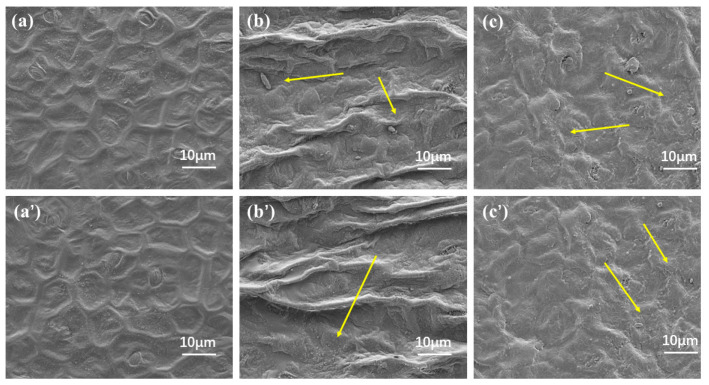
SEM images of cabbage leaf with water and different Avm formulations. (**a**) before and (**a’**) after leaching for water; (**b**) before and (**b’**) after leaching for Avm-EC; (**c**) before and (**c’**) after leaching for Avm@HEMA.

**Figure 9:**
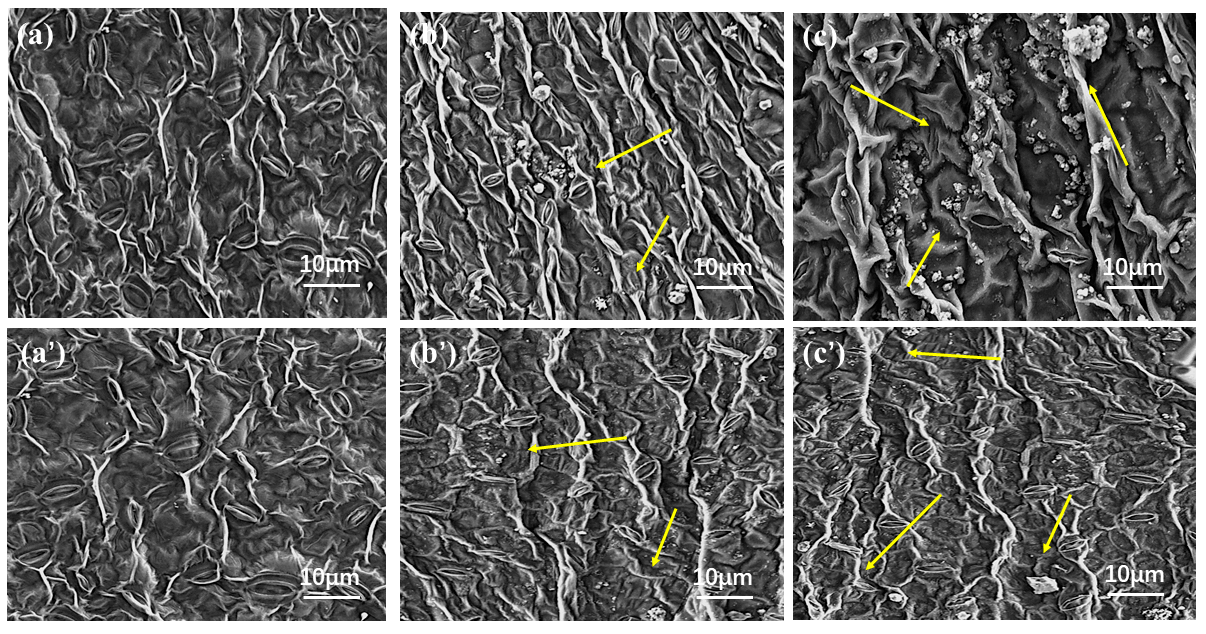
SEM images of cucumber leaf with water and different Avm formulations. (**a**) before and (**a’**) after leaching for water; (**b**) before and (**b’**) after leaching for Avm-EC; (**c**) before and (**c’**) after leaching for Avm@HEMA.

The SEM images of the leaves before and after leaching are shown in Figure 7, Figure 8 and Figure 9. Before leaching, both Avm-EC and Avm@HEMA deposited fine particles on the leaves, and Avm@HEMA deposited more than Avm-EC (Figure 7a–c, Figure 8a–c and Figure 9a–c). After leaching, Avm@HEMA still preserved some solid particles on all the leaves (Figure 7c’, Figure 8c’ and Figure 9c’), while Avm-EC had little particles left (Figure 7a’,b’, Figure 8a’,b’ and Figure 9a’,b’), indicating that Avm@HEMA has stronger foliar adhesion than Avm-EC. After the liquids being sprayed, the droplets spread on the leaf, and the solid particles deposited as the water evaporated. Because the polymer emulsifier itself has adhesive property for it contains a large number of carboxyl groups that could form hydrogen bonds with hydroxyl, carboxyl, and aldehyde groups on the leaf surface [55], the foliar adhesion of the particles could be improved.

The results obtained through the leaf adhesion and retention experiments showed that the adhesion of the Avm@HEMA nanoparticles in the leaf is relatively stronger than that of Avm-EC, thereby increasing the probability of being exposed by insects, so that after entering the insect’s body, it is released in a controlled manner in different internal environments. The Avm@HEMA nanoparticles could stay at the leaf surface or even penetrate into the inner part of plants to improve the insecticidal effects. Therefore, there is no substantial release of this pesticide on the leaves. As the leaves enter the insect’s body via consumption, the nanoparticles begin to exert their effects [56].

### 2.5. Releasing Behavior of Avm@HEMA

**Figure 10:**
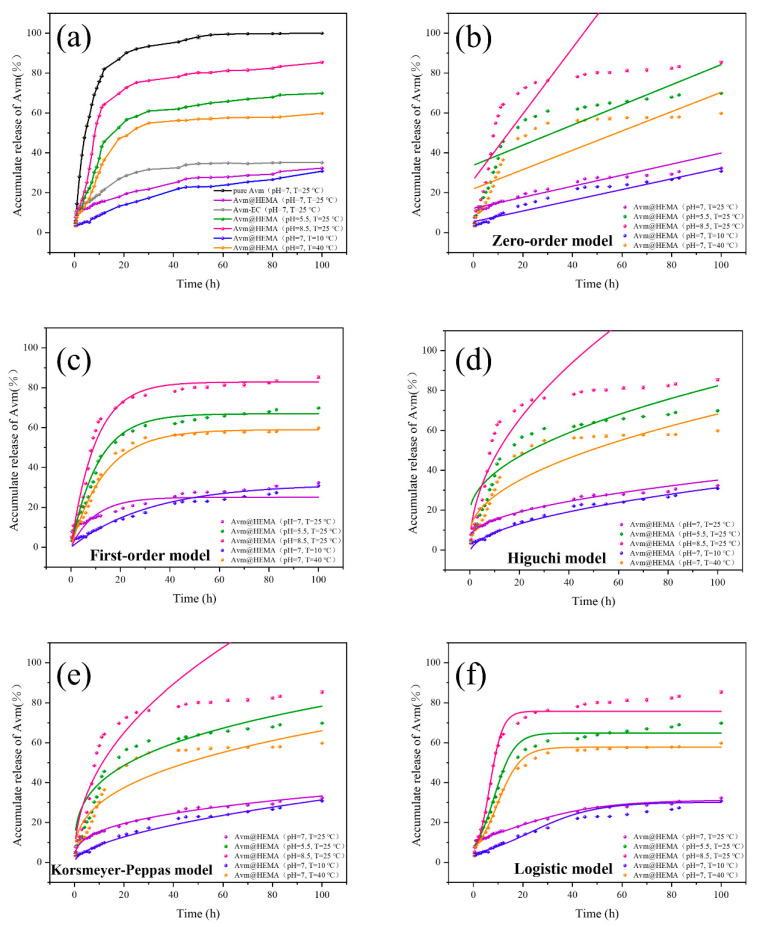
(**a**) The releasing profiles for different Avm formulations under different conditions and fitting the data of the releasing profiles of Avm@HEMA using different kinetic models: (**b**) zero-order model, (**c**) first-order model, (**d**) Higuchi model, (**e**) Korsmeyer–Peppas model, and (**f**) logistic model.

The releasing behavior of Avm-nanoemulsion is another important issue from the application viewpoint. So the releasing process of various samples was investigated by using a UV-Vis spectrometer. Figure 10 shows the releasing profiles of various Avm formulations, including pure Avm, Avm-EC and Avm@HEMA, at different pHs and temperatures in the ethanol/water (1:1, *v*:*v*) mixed solvent. Under normal conditions (pH 7.0 and 25 °C), both Avm-EC and Avm@HEMA show significantly lower releasing rates than pure Avm. Especially, Avm@HEMA has the slowest releasing rate among them with an equilibrium releasing percentage of only around 20% after 80 h.

As indicated in Figure 10, Avm@HEMA displays obvious pH and temperature responsive releasing behaviors. In the fabrication process, Avm molecules were encapsulated in the nanoparticles stabilized by the emulsifier at pH 7.0. However, any change in the pH of the medium may affect the ionizing degree of carboxyl groups as well as the stability of the particles. Therefore, as the pH changes via the introduction of an acid or base, the release of Avm could be accelerated significantly, reaching the equilibrium releasing rate at around 10 h (see Figure 10). The emulsifier molecules underwent protonation and deprotonation at acidic and basic conditions, respectively [55]. At pH 5.5, the nanoparticles became unstable due to the protonation of carboxylate groups, leading to the agglomeration of the particles and the rapid release of Avm molecules. While at pH 8.5, the emulsifier turned out to be more hydrophilic due to deprotonation of carboxyl groups, the particles swelled along with the dissolving of some emulsifier molecules from them, also leading to the rapid releasing of Avm molecules. This assumption was further supported by the fact that the Z-average particle size of Avm@HEMA changed from 556.9 ± 9.0 nm (with PDI 0.569 ± 0.121) at pH 5.5 to 158.9 ± 5.3 nm (with PDI 0.243 ± 0.055) at pH 7.0, and further to 132.4 ± 4.9 nm (with PDI 0.367 ± 0.185) at pH 8.5. Therefore, the more hydrophilicity the emulsifier molecules at a higher pH, the smaller size the nanoparticles should be. Therefore, the changes in the acidity and alkalinity of the medium affect the ionization degree of carboxyl groups as well as the hydrophilic/hydrophobic balance of the emulsifier, promoting the releasing of the Avm molecules from the particles. Practically, as the particles enter the pest’s body via it eating the leaf, small changes in the surrounding medium (acidity and alkalinity) by body fluids will induce a rapid release of Avm molecules from the nanoparticles, which is beneficial to improving the poison’s killing effect.

Moreover, as the temperature increased to 40 °C, the releasing of Avm could also be accelerated obviously, reaching an equilibrium releasing percentage close to 100% within 20 h (see Figure 10). Usually, as the temperature increases, the surface tension of a solvent rapidly decreases, decreasing the interaction between solvent molecules and promoting the diffusion and dissolving of the solute molecules. Therefore, at the high temperature, the faster movement, increased solubility, and reduced solvent viscosity promoted the dissolving of both the emulsifier and Avm from the particles into ethanol/water mixed solvent, reaching the equilibrium releasing state in a shorter period. Typically, the growth and development rate of pests is directly proportional to the temperature within the effective temperature range. The higher the temperature, the faster the development rate. So, the rapid releasing of Avm from the nanoparticles at high temperature should be favorable for inhibiting the growth of pests. In summary, the obtained Avm-namoemulsion exhibited pH and temperature dual responsive releasing features, facilitating the poison’s killing effect.

On the basis of the releasing profiles in Figure 10a, the release process can be divided into three stages: (1) the rapid release from 0–12 h, (2) the slow release from 12–60 h, and (3) the very slow release to reach equilibrium from 60–100 h. To understand the release features of Avm@HEMA under different conditions, typical releasing kinetic models—including zero-order, first-order, Higuchi, Korsmeyer–Peppas, and logistic models [57,58]—were applied to fit the data of the cumulative releasing profiles in Figure 10a, with the results shown in Figure 10b–f and Table 3. When using R-square to define the fitting effect, the Korsmeyer–Peppas model was found to be the most suitable one for the releasing profiles of Avm@HEMA at pH 7.0 and 25 °C, and at pH 7.0 and 10 °C, while Logistic model was more appropriate for the other conditions (pH 5.5 and 25 °C, pH 8.5 and 25 °C, and pH 7.0 and 40 °C), as indicated in Table 3. At pH 7.0 and 25 °C, the values of K_1_ are all smaller than 0.43, illustrating that the releasing behavior of Avm@HEMA was controlled by Fickian diffusion and the single-releasing mode of an exponentially decreasing concentration gradient from the center of the particle to the surrounding medium during the releasing of Avm. At pH 7.0 and 10 °C, the values of K_1_ are between 0.43 and 0.85, illustrating that the releasing behavior of Avm@HEMA at different temperatures is controlled by non-Fickian diffusion (anomalous transport) [59]. The releasing kinetics of Avm@HEMA at different conditions should be helpful for understanding the releasing process and optimizing the formulation of nanopesticides.

In summary, the amphiphilic emulsifier HEMA-3 was able to emulsify highly hydrophobic Avm into the nanoemulsion. Because of the emulsifier, the optimal Avm-nanoemulsion could exhibit a series of desired characters, including high stability, ultraviolet resistance, high leaf spreading and retention, pH and temperature response releasing behavior, which is conducive to the effective use of pesticides. This investigation focused on the fabrication, physical and chemical properties of the Avm-nanoemulsion, which should be helpful for designing more advanced emulsifiers of nanopesticide systems. In the subsequent work, experiments on the field application, the ecotoxicity and the impact on the growth of selected plants of the present nanopesticides will be carried out.

## 3. Materials and Methods

### 3.1. Materials

Ethylene–maleic anhydride copolymer (EMA, prepared in our lab according to reference [60] number average molecular weight (M_n_) is 5978 g/moL, weight average molecular weight (M_w_) is 10,913 g/moL, polydispersity index (PDI) is 1.826), avermectin (Avm, MYM Biological Technology Company, Telangana, IN, USA, 95%), avermectin emulsifiable concentrate (Avm-EC, Chinese Academy of Agricultural Sciences, Beijing, China, 5%), Nile red (Shanghai Bide Pharmatech Co., Ltd., Shanghai, China, 98%), dialysis bag (MD3500, Spectrumlabs, San Francisco, CA, USA). Tetrahydrofuran (THF), dimethyl sulfoxide (DMSO), *N*, *N*-dimethylformamide (DMF), acetonitrile, *N*-methyl-2-pyrrolidinone (NMP), and 1,3-dimethyl-3,4,5,6-tetrahydro-2(^1^H)-pyrimidinone (DMPU) were purchased from Shanghai Titan Scientific Co., Ltd., Shanghai, China, AR. Hydrochloric acid (0.0998 mol/L) and sodium hydroxide (NaOH, 0.0998 mol/L) were purchased from Sigma–Aldrich, St. Louis, MO, USA, AR. Triethylamine (TEA) and phosphotungstic acid were purchased from Shanghai Macklin Biochemical Co., Ltd., Shanghai, China, AR.

### 3.2. Hydrophilic Modification of EMA

According to the literature [61,62], EMA (0.5 g, 4 mmol of acidic anhydride unit) was dissolved in anhydrous THF (10 g), heated, and stirred for 30 min in a three-necked reflux flask. After that, an appropriate amount of deionized water, NaOH (0.35 g, 8.7 mmol) as a reactant or hydrochloric acid (1 g) as a catalyst was added, refluxing at 80 °C for 1 h to obtain an orange–yellow sample solution (20 g). After vacuum-drying to remove the organic solvent and water (100 KPa, 60 °C), orange–yellow solids were obtained, which were labeled as HEMA-1 and HEMA-2, respectively. HEMA-2 was then neutralized by triethylamine (TEA), and the result was defined as HEMA-3.

### 3.3. Fabrication of Avm-Nanoemulsion

The Avm-nanoemulsions were prepared by the phase inversion emulsification process [27,30]. In short, HEMA-1 or HEMA-3 (0.125 g, 1 mmoL) was dispersed in a certain amount (0.5–4 g) of oil phase solvent (NMP, acetonitrile, DMF, DMSO or DMPU). After that, different amounts of Avm were added to form the oil phase. Then the oil phase was added slowly (1.5 mL/min) to an appropriate amount (5–50 g) of the deionized water under moderate stirring. After stirring for 30 min at 600 rpm at room temperature, the resulting sample was dialyzed for 24 h, in which period the water was changed every hour to remove free pesticides and organic solvents, forming the Avm-nanoemulsion.

### 3.4. Characterization of EMA and the Modified EMA

The structures of EMA, HEMA-1, and HEMA-2 were analyzed by Fourier transform infrared spectrometer (FT-IR, Nicolet 560, Thermo Fisher Scientific, Waltham, MA, USA), nuclear magnetic resonance spectrometer (^1^H NMR, JNM-ECZ400S, JEOL, Tokyo, Japan), gel permeation chromatography (GPC, Agilent 1260, Agilent, Santa Clara, CA, USA) and elemental analyzer (EA, Vario EL III, Elementar, Frankfurt, Germany). The critical micelle concentration (CMC) of HEMA-1 and HEMA-3 in water was determined by fluorescence spectrophotometer (RF-5301PC, Shimadzu, Kyoto, Japan). In brief, the test tubes were filled with a certain amount of pyrene solution in acetone, and the acetone was evaporated mostly. Then, the tubes containing pyrene were introduced to the aqueous solutions of emulsifiers at various concentrations (0.01–100 g/L). Fluorescence spectroscopy was used to calculate the CMC of the emulsifier solution after these tubes were shaken for 4 h.

### 3.5. Characterization of Avm-Nanoemulsion

#### 3.5.1. Particle Size and Particle Size Distribution

The Z-average particle size, PDI, and Zeta potential of Avm-nanoemulsions were measured using dynamic light scattering (DLS) instrument (3000HS Zetasizer, Malvern, Malvern, UK). A four-sided cuvette was filled with deionized water, followed by the addition of 4 mL Avm-nanoemulsion. Subsequently, the cuvette was positioned inside the instrument and subjected to the measurement.

#### 3.5.2. Morphology

The morphology of the Avm nanoparticles was observed under a transmission electron microscope (TEM, Hitachi H600, Hitachi, Tokyo, Japan) operating at an accelerating voltage of 80 KV. Based on the diameters of 200 particles that were enumerated from 10 randomly chosen locations in 10 separate TEM images, particle size and size distribution were determined. In addition, confocal laser scanning microscopy (CLSM, ECLIPSE Ti2, Nikon, Tokyo, Japan) was obtained at the excitation wavelength of 530 nm (Nile red).

#### 3.5.3. Solid Content, Drug Loading, and Encapsulation Efficiency

Solid content (SC, %): 0.5 g (M_1_) Avm-nanoemulsions was taken at 60 °C and dried in a vacuum oven until the mass no longer changed to obtain M_2_. The solid content was calculated from the following equation:

$$\mathrm{SC}\;(\%)=\frac{M_{2}}{M_{1}}\times 100\%$$

wherein M_1_ is the mass of Avm-nanoemulsion and M_2_ is the mass of dried Avm-nanoparticles.

The encapsulation efficiency (EE, %) and the drug loading capacity (DLC, %) were determined as follows: 0.5 g Avm-nanoemulsion was added in a centrifuge tube, and the tube was centrifugated at 10,000 rpm for 15 min to remove any uncapsulated Avm. Then, the remaining Avm-nanoemulsion was treated using ultrasound instrumerment for 1 h to extract the encapsulated Avm. Then, the absorbance was measured using a UV–Vis spectrometer (UV-3200, Mapada Instruments, Shanghai, China), and the amount of encapsulated Avm (M_3_) was calculated by the standard curve (A = 0.03323 C + 0.09497, R^2^ = 0.99) at λ=245 nm. The encapsulation efficiency (EE, %) and the drug loading capacity (DLC, %) were calculated from the following equations:

$$\mathrm{EE}\;(\%)=\frac{M_{3}}{M_{0}}\times 100\%$$

$$\mathrm{DLC}\;(\%)=\frac{M_{3}}{M_{4}}\times 100\%$$

wherein M_0_ is the total mass of Avm, M_3_ is the mass of Avm loaded in Avm-nanoparticles, and M_4_ is the mass of Avm-nanoparticles.

#### 3.5.4. Stability

The centrifugal stability, physical stability, dilution stability, storage stability, and photostability of Avm-nanoemulsion were tested as below.

(1) Centrifugal stability. The Avm-nanoemulsions were separated and transferred to identical centrifuge tubes. Z-average particle size, PDI, and Zeta potential in the supernatant were measured using a dynamic light scattering (DLS) instrument (3000HS Malvern Zetasizer) after centrifugation at 10,000 rpm for 3, 6, 9, 12, 15, 18, 21, 24, 27, and 30 min.
(2) Physical stability. According to GB/T 19137-2003 [63], NY/T 1427-2016 [64], and GB/T 19136-2003 [65], the samples were stored at 0 ± 2 °C, 25 ± 2 °C, and 54 ± 2 °C for 21 days, respectively, and the Z-average particle size, PDI, and Zeta potential of the samples were determined every 3 days.
(3) Dilution stability. Avm-nanoemulsions were diluted 20 times (World Health Organization standard), 200 times (GB/T 1603-2001 [66]), and 2000 times (Beijing Green Agricultural Science and Technology Group; active ingredient content: 1.8%, diluting 2000-fold) using deionized water and the Z-average particle size, PDI, and Zeta potential were measured.
(4) Storage stability. The Avm-nanoemulsion was placed in T = 25 °C (T represents temperature) for one month and then the Z-average particle size, PDI, and Zeta potential were tested.
(5) Photostability. Pure Avm and Avm-nanoemulsion containing 10 mg Avm was weighed and mixed with ethanol into a 100 mL glass bottle. After vacuum drying, they were exposed to a 400 W UV lamp (E_max_ = 365 nm) at a distance of 20 cm for 20 min. Dried samples were removed and dissolved in ethanol at 2 min intervals and analyzed by UV–Vis spectrophotometer (UV–Vis) to determine the concentration of undecomposed Avm. The concentration of Avm was measured using a UV-Vis Spectrometer (UV-3200, Mapada Instruments) and calculated by the standard curve (A = 0.03323 C + 0.09497, R^2^ = 0.99) at λ=245 nm.

#### 3.5.5. Surface Tension, Contact Angle, Retention, and Leaf Adhesion

(1) Surface tension. The pendant drop method was used to measure the surface tension of Avm-nanoemulsion by a series of aqueous solutions of emulsifier with a concentration gradient (0.001–100 g/L), and the surface tension curves were measured by a surface tension meter (DCAT21, dataphysics, Filderstadt, Germany).
(2) Contact angle. Fresh plant leaves were collected and fixed on clean slides to avoid damaging the leaf structure and keep the leaves in their natural state. Using water as a blank control, 10 μL of Avm-EC and Avm-nanoemulsion (field use concentration 0.02%) was applied to the rice, cabbage, and cucumber leaves via microinjector drops with a video optical contact angle measuring instrument (OCA 25). After 30 s of dropping, the droplets were photographed on the leaves with the CCD camera on the contact angle meter, and the contact angle of the droplets on the experimental leaves was calculated by a five-point fit analysis. Given the data reliability and complexity of leaf surfaces, measurements were repeated at least five times, and the average values were reported.
(3) Leaf retention and leaching loss. The leaves were immersed in deionized water for 20 min after an ultrasonic cleaning instrument was applied to separate the attached dust, washed three times, and dried. Using water as a blank control, retention and adhesion of Avm-EC and Avm-nanoemulsion (field use concentration 0.02%) on plant leaf surfaces were measured by the Wilhelmy [67] methodl leaf area was measured with a handheld leaf area meter (S, cm^2^). The liquid sample and slender-tipped forceps were placed in a beaker and weighed on a balance, which was then zeroed. A small piece of leaf was fully immersed into the test solution for 30 s and then removed vertically and hang on to the liquid until no droplets drip off. The leaf was then set aside, the forceps were placed back into the beaker and the balance reading W_1_ (g) was recorded. The leaf retention was calculated as R_1_ = W_1_/S (mg/cm^2^). Then, to test the leaching loss, after evaporation of the solvent, the leaves were washed with a continuous stream of water for 0.5 h and again dried naturally for 20 min, at which point the leaves were weighed and their mass recorded as W_2_. The leaf retention was calculated as R_2_ = W_2_/S (mg/cm^2^). Finally, based on the leaf retention before and after leaching, the leaching loss was calculated by 100 × (R_1_ − R_2_)/R_1_ (%). On the other hand, the leaf images were recorded with a field emission scanning electron microscope (FESEM, JSM-7900F, JEOL, Tokyo, Japan) at an accelerating voltage of 5 KV to observe the leaf surface before and after leaching.

#### 3.5.6. Releasing Behavior

The sustained releasing profiles of Avm at different temperatures and pH values were determined following the dynamic dialysis method [68]. For example, 10 mg Avm was loaded into a dialysis bag with 10 mL ethanol/water (1:1, *v*:*v*) and the bag was placed in a wide-necked brown wide-mouth jar with 90 mL of an ethanol/water (1:1, *v*:*v*) for triggering the releasing of Avm. The wide-mouth vials were placed in a constant-temperature shaker (HZ-9120K), releasing at a shaking rate of 150 rpm. At specific time intervals, 4 mL of the solution outside the dialysis bag in the wide-mouth flask was used to measure the Avm concentration and replaced with the same volume of fresh ethanol/water (1:1, *v*:*v*) mixture. A standard curvilinear equation (A = 0.02712 C, R^2^ = 0.99) was obtained by measuring the absorbance at λ=245 nm using a UV–Vis spectrometer (UV-3200, Mapada Instruments) to calculate the concentration of Avm in the mixed solution, which in turn led to the cumulative releasing rate of Avm to evaluate its releasing performance, calculated as follows:

$$E_{r}=\frac{{V_{0}C_{n}+V}_{e}\sum_{1}^{n-1}C_{i}}{m_{drug}}\times 100\%$$

wherein E_r_ is cumulative drug releasing rate, V_e_ and V_0_ represent the volume of displaced solution (4 mL) and the whole volume of the releasing medium (100 mL), C_i_ is the concentration of Avm at the ith replacement sample, m_drug_ is the total mass of Avm in Avm formulation, and n is the number of displacements.

### 3.6. Statistical Analysis

Unless otherwise reported, pH = 7.0, T = 25 °C, all measurements of this article were performed in triplicate, and the results were expressed as mean ± standard deviation (mean ± SD). Data were statistically analyzed by one-way analysis of variance (ANOVA) followed by a Duncan test (*p* < 0.05) using Statistical Product and Service Solutions (SPSS 2022).

## 4. Conclusions

In summary, the amphiphilic polymer emulsifiers, hydrolyzed EMA, were prepared in high hydrolysis degrees via facile one-step processes. With these emulsifiers, pH- and temperature-responsive Avm-nanoemulsions were successfully fabricated by the phase inversion emulsification process. On the basis of RSM, the optimal formulation of Avm-nanoemulsion was selected in terms of the particle size, encapsulation rate and drug loading. Under the optimal formulation, the obtained Avm-nanoemulsion Avm@HEMA showed a series of desired performances, including good stability and UV resistance, low surface tension, high leaf spreading, and retention on various leaves. Especially, Avm@HEMA displayed pH and temperature dual responsive releasing features. Under normal conditions (at pH 7.0 and 25 °C), the releasing rate of Avm was rather low, but it significantly increased under either weak acids or weak base, or at high temperature. Korsmeyer-Peppas model was the most suitable one for the releasing profiles of Avm@HEMA at pH 7.0 and 25 °C (controlled by Fickian diffusion) as well as pH 7.0 and 10 °C (controlled by non-Fickian diffusion), while Logistic model was appropriate for the other conditions (pH 5.5 and 25 °C, pH 8.5 and 25 °C, pH 7.0 and 40 °C). This investigation has demonstrated that all the excellent performances of Avm@HEMA could be attributed to the emulsifiers, which should be helpful for designing advanced polymer emulsifiers of nanopesticide delivery systems. For further improve the stability of nanopesticide, novel polymer emulsifiers should be designed and fabricated with a desired molecular size and well distributed functional groups along the chains.

## Figures and Tables

**Table 1:** Mn, Mw, PDI, and element contents of EMA, HEMA-1, and HEMA-2.

Sample	Mn (g/mol)	Mw (g/mol)	PDI	C (%)	H (%)	O (%)
EMA	5978	10,913	1.83	54.81 ± 0.66	4.15 ± 0.23	40.63 ± 0.22
HEMA-1	8623	17,619	2.04	44.97 ± 0.56	5.95 ± 0.20	45.26 ± 0.42
HEMA-2	7680	14,502	1.89	44.52 ± 0.44	5.67 ± 0.10	45.14 ± 0.37

**Table 2:** Properties of Avm-nanoemulsions using HEMA-1 and HEMA-3 as the emulsifiers.

Emulsifier	Z-Average Particle Size/nm	PDI	Zeta Potential/mV
HEMA-1	563.1 ± 22.5	0.109 ± 0.102	−32.0 ± 1.0
HEMA-3	231.0 ± 3.4	0.069 ± 0.053	−50.4 ± 1.5

**Table 3:** Fitting results for the releasing curves of Avm@HEMA.

Releasing Model	Releasing Condition	Parameter			
		k_1_	k_2_	k_3_	R^2^
Zero-order y = k_1_t + k_2_	pH = 7.0, T = 25 °C	0.003	0.122		0.903
	pH = 8.5, T = 25 °C	0.005	0.337		0.584
	pH = 5.5, T = 25 °C	0.017	0.265		0.637
	pH = 7.0, T = 10 °C	0.003	0.055		0.941
	pH = 7.0, T = 40 °C	0.005	0.219		0.718
First-order y = k_2_[1 − exp(−k_1_t)]	pH = 7.0, T = 25 °C	0.102	0.251		0.741
	pH = 8.5, T = 25 °C	0.092	0.669		0.982
	pH = 5.5, T = 25 °C	0.101	0.828		0.936
	pH = 7.0, T = 10 °C	0.031	0.317		0.986
	pH = 7.0, T = 40 °C	0.073	0.590		0.992
Higuchi y = k_1_$t^{1/2}$+ k_2_	pH = 7.0, T = 25 °C	0.029	0.003		0.985
	pH = 8.5, T = 25 °C	0.064	0.032		0.770
	pH = 5.5, T = 25 °C	0.146	0.050		0.796
	pH = 7.0, T = 10 °C	0.033	0.002		0.992
	pH = 7.0, T = 40 °C	0.060	0.029		0.881
Korsmeyer-Peppas y = k_2_$t^{k_{1}}$	pH = 7.0, T = 25 °C	0.333	0.072		0.995
	pH = 8.5, T = 25 °C	0.297	0.200		0.855
	pH = 5.5, T = 25 °C	0.460	0.164		0.803
	pH = 7.0, T = 10 °C	0.558	0.024		0.994
	pH = 7.0, T = 40 °C	0.353	0.130		0.917
Logistic $y=\frac{k_{3}}{1+exp[-k_{2}(t-k_{1})]}$	pH = 7.0, T = 25 °C	12.315	0.054	0.313	0.992
	pH = 8.5, T = 25 °C	8.324	0.243	0.649	0.983
	pH = 5.5, T = 25 °C	6.574	0.382	0.758	0.995
	pH = 7.0, T = 10 °C	23.037	0.077	0.302	0.989
	pH = 7.0, T = 40 °C	9.594	0.212	0.577	0.995

## Data Availability

Data are contained within the article and Supplementary Materials.

## Supplementary Materials

The following supporting information can be downloaded at: https://www.mdpi.com/article/10.3390/molecules29051148/s1, Figure S1. ^1^H NMR spectra of (a) EMA, (b) HEMA-1, and (c) HEMA-2. Figure S2. Local magnification ^1^H NMR spectrum of EMA. The values under the peaks indicate the peak areas. Table S1. Experimental matrix of Avm-nanoemulsion based on central composite design (CCD). Figure S3. Response surface and contour plots of the interactions between (a) A vs. B, (b) A vs. D, and (c) C vs. D on the EE of Avm-nanoemulsion. Figure S4. Response surface and contour plots of the interactions between (a) A vs. B, (b) A vs. C, (c) A vs. D, (d) B vs. C, (e) B vs. D, and (f) C vs. D on the DLC of Avm-nanoemulsion. Figure S5. Confocal laser scanning microscopic image of Avm@HEMA with Nile red labeling. Figure S6. (a) Appearance, Z-average particle size, PDI, and Zeta potential of Avm@HEMA before and after dilution, (b) appearance, Z-average particle size, PDI, and Zeta potential of Avm@HEMA before and after storage for one month. Table S2. The Z-average particle size, PDI, Zeta potential, encapsulation efficiency (EE) and drug loading capacity (DLC) over time of Avm@HEMA. Figure S7. Remaining percentage of Avm at different irradiation time. Figure S8. Surface tension at different concentrations of the HEMA-3 aqueous solution. Figure S9. Contact angles of deionized water, Avm-EC, and Avm@HEMA on rice, cabbage, and cucumber leaves. (Values not sharing a common letter within the same row in each element indicate a significant difference, *p* < 0.05).
