# Supplementary material for: Fabrication of Eco-Friendly Hydrolyzed Ethylene–Maleic Anhydride Copolymer–Avermectin Nanoemulsion with High Stability, Adhesion Property, pH, and Temperature-Responsive Releasing Behaviors
Source: Molecules. 2024 Mar 5;29(5):1148. doi: 10.3390/molecules29051148 (PMC10934311; doi:10.3390/molecules29051148)
Supplement: Supplementary file 1 [file molecules-29-01148-s001.zip › molecules-2861730-supplementary.pdf]

Fabrication of Eco-Friendly Hydrolyzed Ethylene–Maleic Anhydride Copolymer–Avermectin Nanoemulsion with High Stability, Adhesion Property, pH, and Temperature-Responsive Releasing Behaviors

Yuxin Cheng, Zeyu Pan, Liming Tang *, Yanbin Huang and Wantai Yang

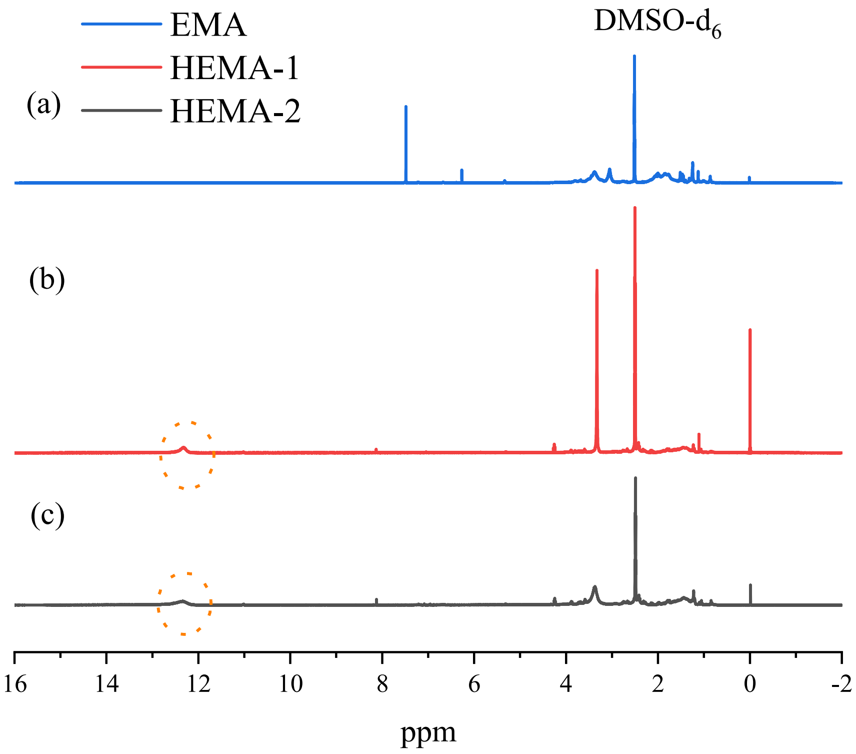

Figure S1. ^{1}H NMR spectra of (a) EMA, (b) HEMA-1, and (c) HEMA-2.

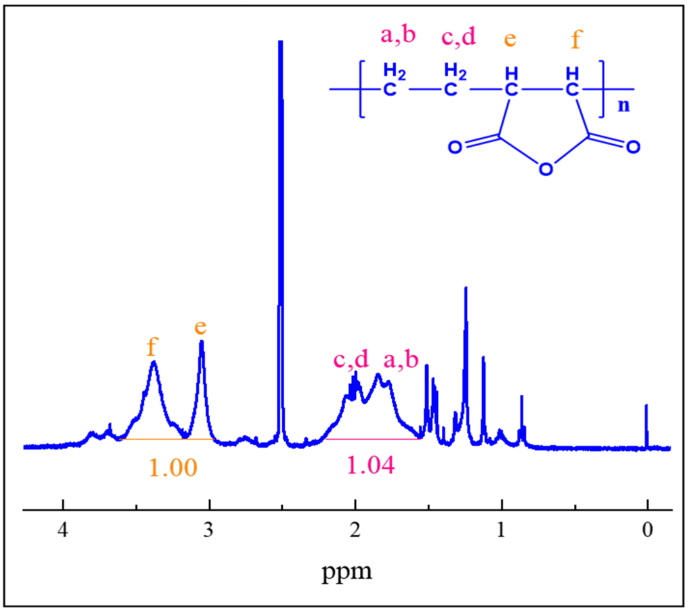

Figure S2. Local magnification ^{1}H NMR spectrum of EMA. The values under the peaks indicate the peak areas.

Table S1. Experimental matrix of Avm-nanoemulsion based on central composite design (CCD).

Run	Independent Variables				Response Variables					
	A (%)	B (%)	C (g)	D	Y_1 (nm)		Y_2 (%)		Y_3 (%)	
					Mean	standard deviation	Mean	standard deviation	Mean	standard deviation
1	33.3	0.47	0.50	1	298.70	4.0	44.64	2.61	52.52	3.34
2	33.3	2.60	0.50	1	392.50	3.3	50.64	3.50	57.34	3.95
3	66.65	0.47	2.25	3	389.60	3.9	65.72	3.49	57.93	2.61
4	33.3	2.60	4.00	1	278.60	2.6	54.64	2.95	62.52	3.50
5	66.65	0.47	2.25	3	198.80	3.5	69.34	4.01	55.93	3.44
6	33.3	2.60	4.00	1	201.50	3.4	59.64	2.80	69.52	2.95
7	33.3	0.47	0.50	5	325.70	2.9	49.64	4.20	57.23	4.03
8	66.65	2.60	2.25	-1	228.40	4.0	66.72	2.60	54.77	2.80
9	133.35	0.47	2.25	3	230.50	2.8	56.34	3.34	49.23	4.20
10	66.65	2.60	2.25	3	188.80	4.2	67.56	4.55	55.93	3.44
11	100	0.47	0.50	5	352.20	2.6	74.88	4.01	54.93	2.21
12	-0.05	2.60	2.25	3	245.20	3.3	53.21	2.80	54.44	4.13
13	33.3	0.47	4.00	5	234.20	4.5	55.64	4.20	62.34	2.80

14	100	2.60	4.00	1	303.10	4.2	77.88	2.60	57.93	4.20
15	100	0.47	0.50	1	360.30	4.1	74.23	3.34	56.90	2.60
16	33.3	2.60	4.00	5	205.90	3.6	60.6	4.55	67.24	3.87
17	66.65	0.47	2.25	3	157.30	3.0	65.3	4.21	55.33	4.52
18	100	2.60	0.50	5	230.50	4.1	75.8	4.11	59.02	4.21
19	66.65	0.47	1.25	3	333.50	3.7	69.2	3.56	57.23	4.10
20	66.65	2.60	2.25	3	198.80	3.4	68.3	3.07	59.89	4.10
21	66.65	0.47	2.25	3	198.80	4.2	66.4	4.10	56.22	3.60
22	100	2.60	4.00	1	198.40	4.3	74.6	3.70	56.90	3.70
23	66.65	0.47	5.75	3	168.30	2.9	67.3	3.60	54.99	3.49
24	66.65	2.60	2.25	7	202.90	3.5	68.9	3.45	54.34	4.21
25	33.3	0.47	0.50	5	287.60	3.7	45.3	4.10	51.87	3.72
26	100	2.60	0.50	1	235.80	2.4	77.2	3.70	58.21	2.55
27	100	0.47	4.00	5	275.30	3.2	75.2	3.49	57.89	3.56
28	66.65	2.60	2.25	3	198.80	3.2	69.7	4.21	55.90	3.78
29	100	0.47	4.00	5	202.30	4.2	72.3	4.33	56.78	2.75
30	66.65	2.60	0.50	3	198.80	4.0	69.2	2.95	55.67	3.63

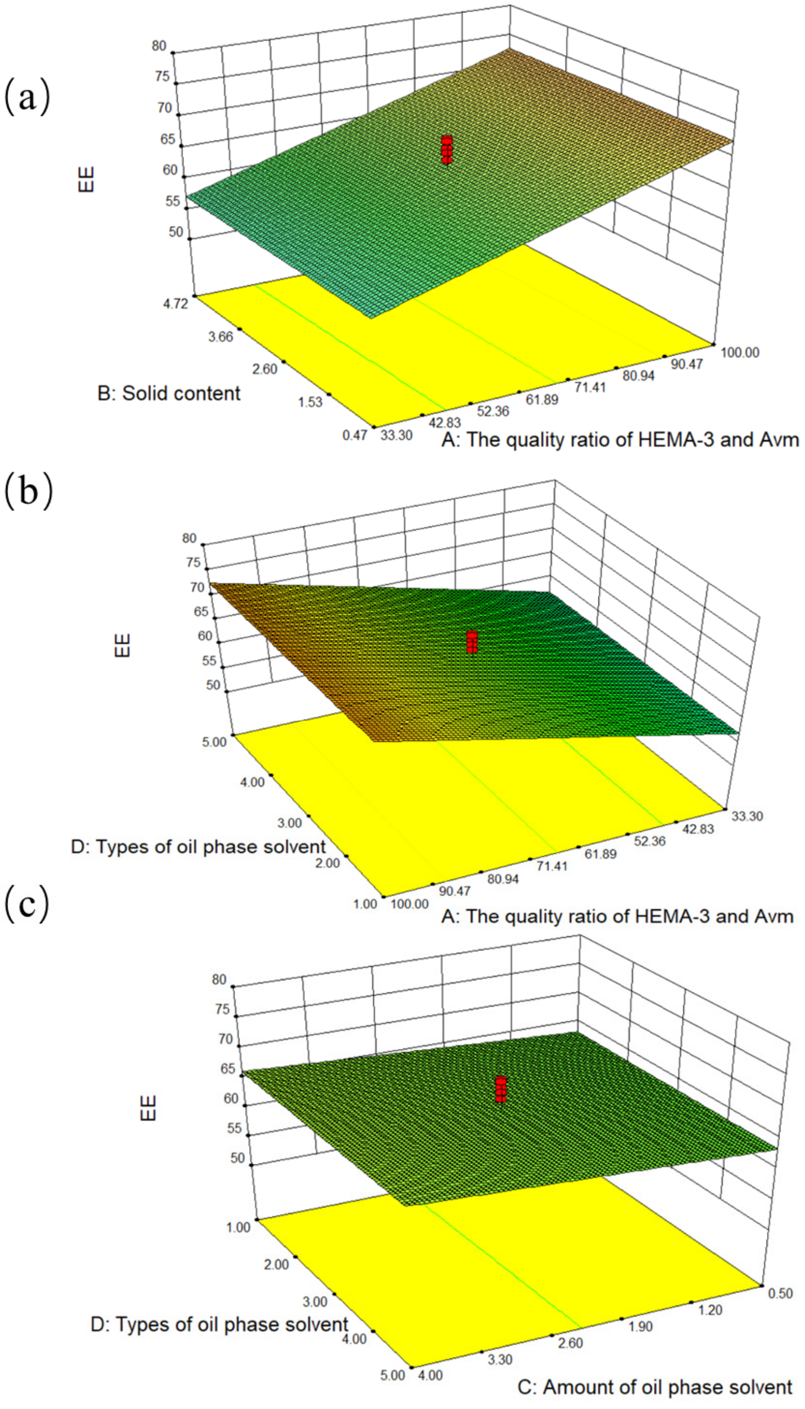

Figure S3. Response surface and contour plots of the interactions between (a) A vs. B, (b) A vs. D, and (c) C vs. D on the EE of Avm-nanoemulsion.

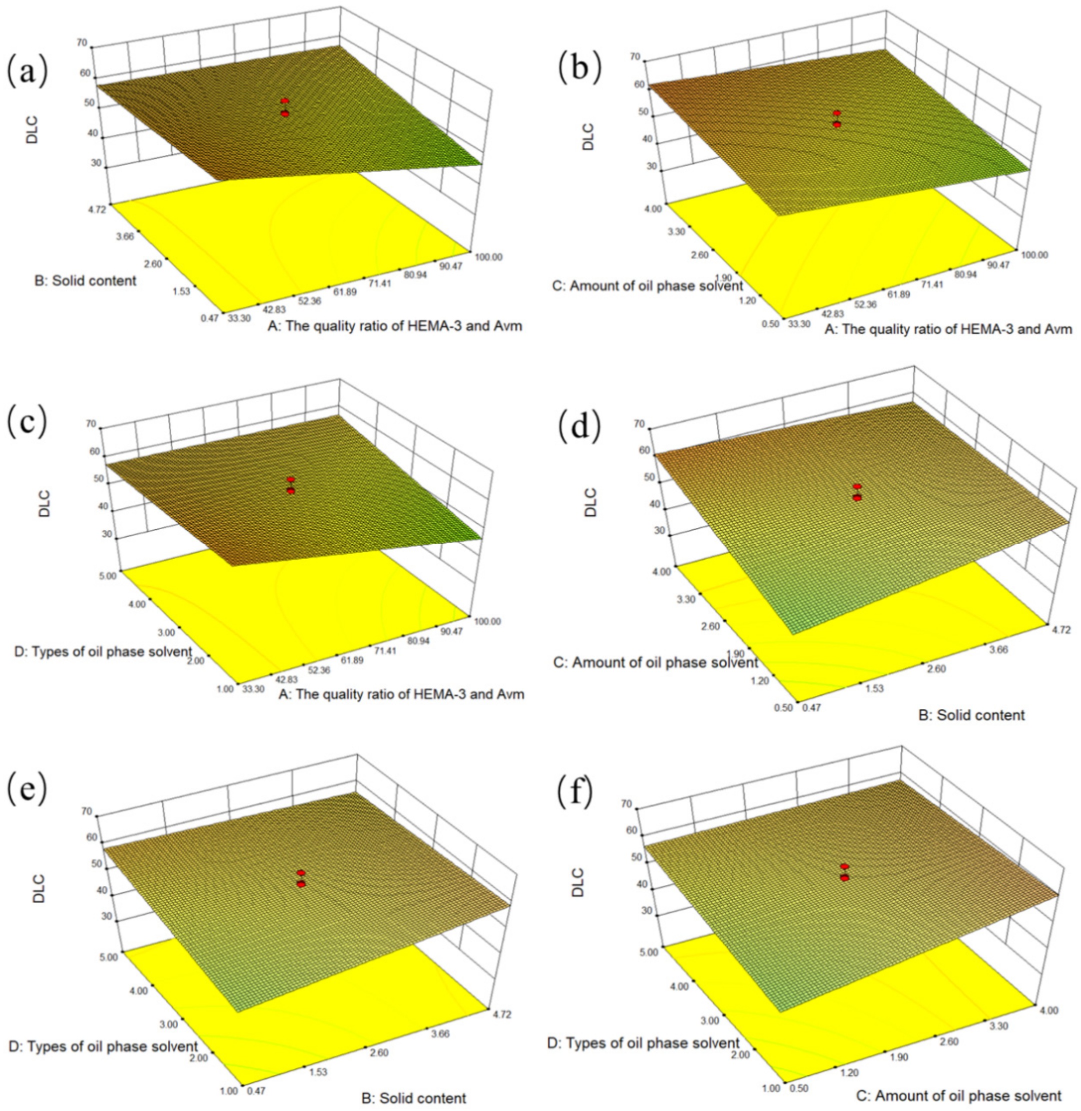

Figure S4. Response surface and contour plots of the interactions between (a) A vs. B, (b) A vs. C, (c) A vs. D, (d) B vs. C, (e) B vs. D, and (f) C vs. D on the DLC of Avm-nanoemulsion.

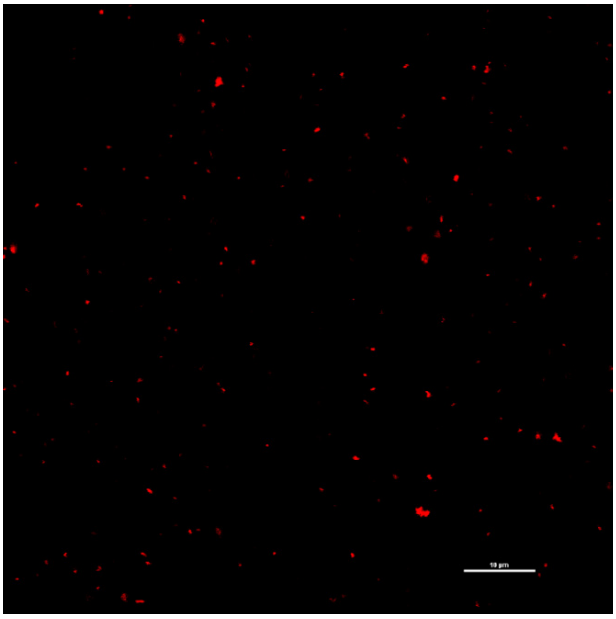

Figure S5. Confocal laser scanning microscopic image of Avm@HEMA with Nile red labeled.

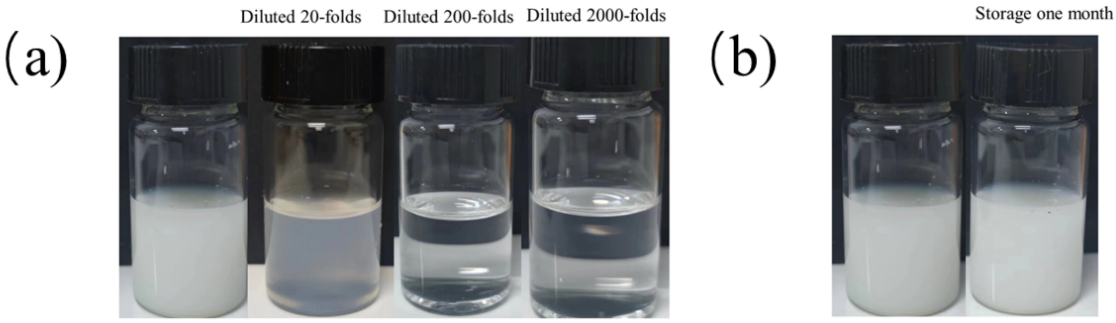

Z-average particle size (nm)	156.8±5.0	160.4±12.5	167.5±3.5	164.1±4.3
PDI	0.078±0.003	0.293±0.132	0.102±0.075	0.092±0.037
Zeta potential (mV)	-49.2±0.9	-51.1±3.5	-34.9±1.6	-31.5±2.2

Z-average particle size (nm)	156.8±5.0	163.7±2.9
PDI	0.078±0.003	0.133±0.021
Zeta potential (mV)	-49.2±0.9	-44.7±0.3

Figure S6. (a) Appearance, Z-average particle size, PDI, and Zeta potential of Avm@HEMA before and after dilution, (b) Appearance, Z-average particle size, PDI, and Zeta potential of Avm@HEMA before and after storage for one month.

Table S2. The Z-average particle size, PDI, Zeta potential, encapsulation efficiency (EE) and drug loading capacity (DLC) over time of Avm@HEMA.

Time/day	Z-average particle size	PDI	Zeta potential	encapsulation efficiency (EE)/%	drug loading capacity (DLC)/%
0	156.8±5.0	0.115±0.003	-47.8±0.9	69.72±4.01	54.93±1.12
3	157.2±3.1	0.143±0.015	-47.9±0.7	68.44±2.03	52.77±2.31
6	158.3±2.8	0.133±0.009	-46.9±0.4	68.44±2.03	52.22±0.53
9	159.0±3.0	0.116±0.012	-44.9±0.6	66.52±2.77	50.54±1.70
12	161.4±4.5	0.118±0.008	-43.9±0.9	63.28±3.05	49.83±1.77
15	159.2±1.0	0.132±0.022	-42.8±0.5	64.43±1.98	51.93±0.79
18	160.7±1.9	0.084±0.021	-41.4±0.3	63.12±2.54	52.54±1.30
21	161.9±2.0	0.078±0.002	-40.8±0.9	65.52±1.09	52.67±1.44
24	162.0±1.0	0.128±0.108	-42.6±0.5	61.28±1.05	51.63±0.55
27	163.4±2.5	0.122±0.012	-45.7±0.3	60.43±1.28	50.89±1.39
30	163.7±2.9	0.133±0.021	-44.7±0.3	60.12±2.54	50.54±1.34

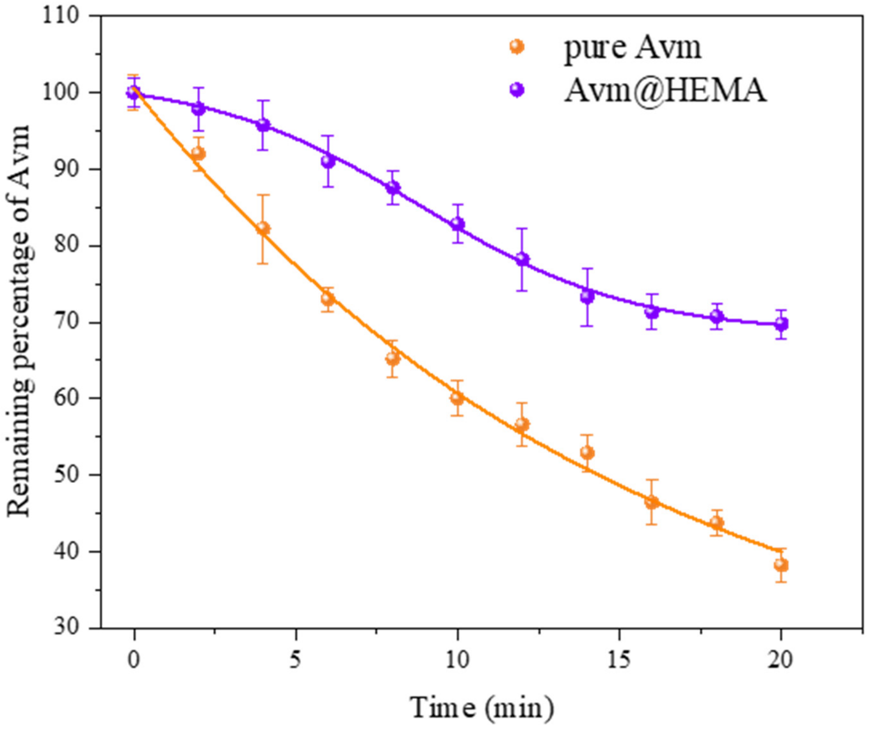

Figure S7. Remaining percentage of Avm at different irradiation time.

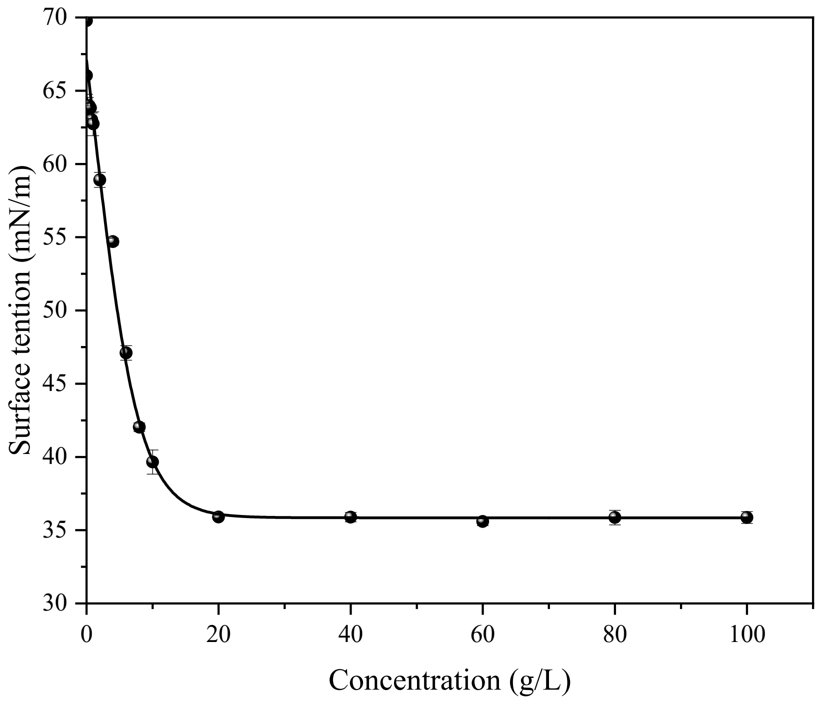

Figure S8. Surface tension at different concentrations of the HEMA-3 aqueous solution.

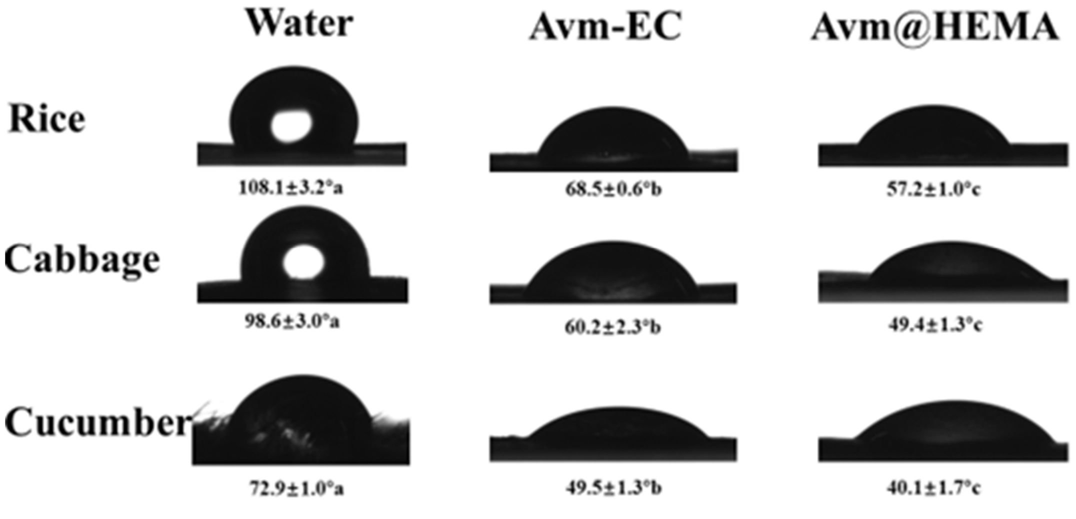

Figure S9. Contact angles of deionized water, Avm-EC, and Avm@HEMA on rice, cabbage, and cucumber leaves. (Values not sharing a common letter within the same row in each element indicate a significant difference, $p < 0.05$).
